# Editorial: Data driven analytics in orofacial pain

**DOI:** 10.3389/fdmed.2026.1959814

**Published:** 2026-08-18

**Authors:** Linda Sangalli, Akhilanand Chaurasia, Maria Bankvall

**Affiliations:** 1 College of Dental Medicine-Illinois, Midwestern University, Downers Grove, IL, United States; 2 Department of Oral Medicine and Radiology, King George's Medical University, Lucknow, India; 3 Centre of Gerodontology, Public Dental Service, Region Västra Götaland, Gothenburg, Sweden; 4 Clinic for Orofacial Medicine, Public Dental Service, Region Västra Götaland, Uddevalla, Sweden

**Keywords:** artificial intellegence, data-driven methodology, epidemiology, orofacial pain, precision medicine

Orofacial pain remains one of the most challenging conditions, given to heterogeneity and overlapping symptomatology, lack of diagnostic testing, reliance on patient's self-reported symptoms, and contribution of biopsychological factors to their onset and chronicity. These clinical challenges are compounded with limited training of general dental providers and primary care providers at the entry-level, insufficient availability of orofacial pain specialists, insurance coverage challenges, and their intersection between medicine and dentistry. As a result, patients often experience delayed diagnosis, fragmented care, multiple specialist consultations, and variable treatment outcomes (1). Research advances in computational analytics, large-scale clinical datasets, systematic evidence synthesis, and digital health technologies are transforming how clinicians and researchers investigate orofacial pain. Instead of relying solely on individual clinical experience, data-driven approaches allow for the identification of prognostic factors, prediction of treatment response, and monitoring of population health trends to generate evidence that can support precision medicine (2). The present Research Topic, *Data Driven Analytics in Orofacial Pain,* brings together four complimentary contributions that illustrate how data-driven methodologies are being applied within orofacial pain research, specifically including evidence synthesis from systematic analyses, prediction modeling, global epidemiology, and image-guided intervention. While these studies address different pain conditions and employ distinct methodologies, altogether they illustrate an ongoing transition in pain research, from describing disease to generating actionable knowledge capable of informing clinical decision-making and precision medicine (3).

In both their studies, Tian et al. and Li et al. highlight the heterogeneity of pharmacological and surgical treatments for trigeminal and glossopharyngeal neuralgia, respectively, that incur in 10%–50% recurrence rates, partial efficacy, and side effects. Given the wide range of treatment success, both papers emphasized the need for clinicians to have better tools to identify patients that most likely will benefit from specific interventions, highlighting the need for a personalized medicine approach. In their systematic review, Tian et al. demonstrated the growing use of prediction models which show reasonable accurate discrimination (area under the curve, AUC > 0.8), although emphasized the need for external validation. In their retrospective analysis of 13 patients, Li et al. demonstrated how modern image-guided intervention, a minimally invasive treatment conducted under ultrasound guidance, produced promising results, providing real-world evidence supporting its safety and therapeutic potential. Using prediction models and standardized outcome reporting, future research should move towards the creation of multicenter registries where machine learning could help identify individualized treatment recommendations based on large data.

Zhong et al. emphasizes the significant challenges of chronic pain management in the medical field, which have shown promising results with advances in the understanding of the multidimensionality of chronic pain mechanisms. Integrating preclinical and clinical evidence, at the mechanistic level of neurotransmitters, descending modulation, and cortical plasticity, with chronic pain conditions help informing personalized treatment. Central sensitization and maladaptive pian processing are shared across many chronic pain disorders, including orofacial pain (4).

The comprehensive analysis of the global, regional, and national burden of headache by Shen et al. emphasize the role of worldwide surveillance and resource allocation, and corresponding inequities, that can inform public health policy. Based on the current trends of global incidence of headache at 809.2 million, headache disorders are expected to surpass 3.5 billion according to 2050 projections. Current and estimated trends call for enhanced prevention, early diagnosis, and multidisciplinary care to inform targeted health policy with the ultimate goal of enhancing patient care and patient-reported outcomes.

Ongoing and future research in data-driven analytics should address several important methodological limitations highlighted by the studies included in this Research Topic. First, there is a need to enhance data quality and harmonization across studies. In this context, the adoption of standardized diagnostic frameworks (e.g., Diagnostic Criteria for Temporomandibular Disorders [DC/TMD], International Classification of Orofacial Pain [ICOP]) together with standardized patient-reported outcome measures, will improve consistency and comparability of findings, and strengthen the development and external validation of prediction models. Likewise, adherence to FAIR (Findable, Accessible, Interoperable, and Reusable) data principles can promote data sharing and reproducibility through multicenter collaborations and secondary analyses (5).

Second, machine learning and artificial intelligence are becoming increasingly relevant for integrating multimodal data derived from imaging, biomarkers, patient-reported outcomes, wearable devices, and electronic health records (6). Such approaches have the potential to improve disease phenotyping, enhance risk stratification, support earlier diagnosis, predict treatment response, and facilitate personalized management strategies (7). Yet these approaches will only become clinically useful if models are transparent, externally validated, and developed using representative patient populations that minimize algorithmic bias (8).

Third, the future of data-driven orofacial pain research will increasingly depend on international and large-scale collaborations. Given that several neuropathic orofacial pain conditions are relatively uncommon and heterogeneous in their clinical presentation, multicenter registries and collaborative research networks can overcome limitations derived from single-institution, small datasets toward increasing generalizability of the findings across populations and facilitating translation of research into clinical practice.

In summary, the contributions in this Research Topic illustrate the evolution of pain research from descriptive epidemiology toward predictive and ultimately prescriptive analytics. Quantitative methods based on larger datasets can improve understanding and management of complex pain disorders and patient outcomes, provided that they are based on standardized methodologies, rigorous validation, and transparent reporting. The value of data-driven analytics will be measured by their ability to improve diagnosis, personalize treatment, reduce healthcare disparities, and enhance quality of life for individuals living with orofacial pain.
